# Reunion of Cut-off Fingers

**Published:** 1892-06

**Authors:** 


REUNION OF CUT-OFF FINGERS.—Gluck ("Deut. med. Woch.") reports the case of a butcher who cut off with a cleaver the terminal phalanges of the left ring and middle fingers. The resultant wound was disinfected and the parts sutured. Perfect union resulted. Similar cases are reported in the *Medical Standard*, Vol. I to X.—(*Ex.*)

